# Suggestions for improving the design of clinical trials in multiple sclerosis—results of a systematic analysis of completed phase III trials

**DOI:** 10.1007/s13167-019-00192-z

**Published:** 2019-11-22

**Authors:** Sinje Gehr, Thomas Kaiser, Reinhold Kreutz, Wolf-Dieter Ludwig, Friedemann Paul

**Affiliations:** 1 Charité Universitätsmedizin Berlin, Charitéplatz 1, 10117 Berlin, Germany; 2 Institut für Qualität und Wirtschaftlichkeit im Gesundheitswesen (Institute for Quality and Efficiency in Health Care) (IQWiG), Im Mediapark 8, 50670 Köln, Germany; 3 Arzneimittelkommission der deutschen Ärzteschaft (Drug Commission of the German Medical Association), Herbert-Lewin-Platz 1, 10623 Berlin, Germany

**Keywords:** Multiple sclerosis, Clinical trial, Phase III, Patient-reported outcome measures (PROM), Comparator, Patient preferences, Personalized provision of medical care, Predictive surrogate measures, Targeted prevention, Predictive preventive personalised medicine, Immunotherapy, Immunomodulatory drugs, Patient stratification, Patient benefits, Risk analysis, Mitigation, Relapse-prevention approach, Fatigue, Depression, Cognitive impairment, Criteria

## Abstract

This manuscript reviews the primary and secondary endpoints of pivotal phase III trials with immunomodulatory drugs in multiple sclerosis (MS). Considering the limitations of previous trial designs, we propose new standards for the planning of clinical trials, taking into account latest insights into MS pathophysiology and patient-relevant aspects. Using a systematic overview of published phase III (pivotal) trials performed as part of application for drug market approval, we evaluate the following characteristics: trial duration, number of trial participants, comparators, and endpoints (primary, secondary, magnetic resonance imaging outcome, and patient-reported outcomes). From a patient perspective, the primary and secondary endpoints of clinical trials are only partially relevant. High-quality trial data pertaining to efficacy and safety that stretch beyond the time frame of pivotal trials are almost non-existent. Understanding of long-term benefits and risks of disease-modifying MS therapy is largely lacking. Concrete proposals for the trial designs of relapsing (remitting) multiple sclerosis/clinically isolated syndrome, primary progressive multiple sclerosis, and secondary progressive multiple sclerosis (e.g., study duration, mechanism of action, and choice of endpoints) are presented based on the results of the systematic overview. Given the increasing number of available immunotherapies, the therapeutic strategy in MS has shifted from a mere “relapse-prevention” approach to a personalized provision of medical care as to the choice of the appropriate drugs and their sequential application over the course of the disease. This personalized provision takes patient preferences as well as disease-related factors into consideration such as objective clinical and radiographic findings but also very burdensome symptoms such as fatigue, depression, and cognitive impairment. Future trial designs in MS will have to assign higher relevance to these patient-reported outcomes and will also have to implement surrogate measures that can serve as predictive markers for individual treatment response to new and investigational immunotherapies. This is an indispensable prerequisite to maximize the benefit of individual patients when participating in clinical trials. Moreover, such appropriate trial designs and suitable enrolment criteria that correspond to the mode of action of the study drug will facilitate targeted prevention of adverse events, thus mitigating risks for individual study participants.

## Background

Since the development of the first immunotherapy interferon beta-1b in 1995 (see Table 1), a number of immunomodulatory substances have been authorized for the disease-modifying treatment of multiple sclerosis (MS), namely by reducing relapse rates [13, 25, 58]. The mechanisms of action have been fully elucidated for only few of these drugs. While a positive effect on the autoreactive, inflammatory immune response is considered proven, direct neuroprotective effects are unlikely.

**Table 1:** Published pivotal phase III trials

Trial start	Approval (year, EU)	Indication trial	Test substance (trade name)	Trial duration, trial participants (N total)	Trial acronym	Comparator drug	Primary endpoints	Further endpoints & measurements	Online sources
1988	1995	RRMS	Interferon-β1b (Betaferon®)	104 weeks, N 372	MSSG	Placebo	REL	D, MRI, UE	[1, 2]
1990	1997	RRMS	Interferon-β1a IM (Avonex®)	104 weeks, N 301	MSCRG	Placebo	D	UE	[1, 6]
1991	2001	RRMS	Glatiramer acetate (Copaxone®)	104 weeks, N 251	CMSSG	Placebo	REL	D, MRI, UE	[1, 10]
1994	1998	RMS	Interferon-β1a SC (Rebif®)	104 weeks, N 560	PRISMS	Placebo	REL	D, O, UE	[1, 7]
1996	1997	CIS	Interferon-β1a SC (Avonex®)	78 weeks, N 383	CHAMPS	Placebo	O, D	MRI, PROM, UE	[33]
1997	1995	RRMS	Interferon-β1b (Betaferon®)	104 weeks, N 188	INCOMIN	Interferon β-1a IM	REL	D, MRI, UE	[1, 4]
2001	2006	RMS	Natalizumab (Tysabri®)	104 weeks, N 942	AFFIRM	Placebo	REL	MRI, D, UE	[1, 13]
2002	1995	CIS	Interferon-β1b (Betaferon®)	104 weeks, N 468	BENEFIT	Placebo	O, REL, D	MRI, UE	[5]
2003	1995	RRMS	Interferon-β1b (Betaferon®)	104 weeks, N 2244	BEYOND	Glatiramer acetate	REL	D, MRI, O, UE	[1, 3]
2004	1998	RMS	Interferon-β1a SC (Rebif®)	96 weeks, N 766	REGARD	Glatiramer acetate	REL	MRI, D, UE	[1, 8]
2004	2001	CIS^a^	Glatiramer acetate (Copaxone®)	156 weeks, N 481	PRECISE	Placebo	O	MRI, UE	[11]
2004	2013	RMS	Teriflunomide (Aubagio®)	108 weeks, N 1088	TEMSO	Placebo	REL	D, O, MRI, PROM, UE	[1, 36, 20]
2005	2017	RMS	Cladribine (Mavenclad®)	96 weeks, N 1326	CLARITY	Placebo	REL	D, MRI, UE	[27, 28]
2006	1998	CIS	Interferon β-1a (Rebif®)	104 weeks, N 517	REFLEX	Placebo	O	UE	[31, 32, 34]
2006	2011	RMS	Fingolimod (Gilenya®)	104 weeks, N 1272	FREE-DOMS	Placebo	REL	D, MRI, O, UE	[1, 14, 35]
2006	2011	RRMS	Fingolimod (Gilenya®)	104 weeks, N 1083	FREE-DOMS 2	Placebo	REL	MRI, D, PROM, UE	[1, 35, 15]
2006	2011	RMS	Fingolimod (Gilenya®)	52 weeks, N 1292	TRANS-FORMS	Interferon β-1a i.m.	REL	MRI, D, UE	[1, 35, 16]
2007	2013	RRMS	Alemtuzumab (Lemtrada®)	104 weeks, N 581	CARE MS 1	Interferon β-1a SC	REL, D	MRI, O, UE	[1, 20]
2007	2013	RMS	Alemtuzumab (Lemtrada®)	104 weeks, N 840	CARE MS 2	Interferon β-1a SC	REL, D	MRI, O, UE	[1, 21]
2007	2014	RMS	Dimethyl fumarate (Tecfidera®)	96 weeks, N 1234	DEFINE	Placebo	REL	MRI, D, UE	[1, 22]
2007	2014	RRMS	Dimethyl fumarate (Tecfidera®)	96 weeks, N 1417	CONFIRM	Placebo/Glatiramer acetate	REL	MRI, D, O, UE	[1, 23]
2008	2013	RMS	Teriflunomide (Aubagio®)	48 weeks, N 1169	TOWER	Placebo	REL	D, O, PROM, UE	[1, 17, 36]
2009	2013	RMS	Teriflunomide (Aubagio®)	96 weeks, N 324	TENERE	Interferon-β 1a SC	REL	PROM, UE	[1, 36, 19]
2009	2014	RRMS	Peginterferon-1A (Plegidry®)	104 weeks, N 1516	ADVANCE	Placebo	REL	MRI, D, UE	[1, 24]
2010	2016	RRMS	Daclizumab (Zinbryta®)	144 weeks, N 1841	DECIDE	Interferon β-1a	REL	MRI, PROM, UE	[25, 26]
2011	2018	RMS	Ocrelizumab (Ocrevus®)	96 weeks, N (I) 821 + N (II) 835	OPERA I OPERA II	Interferon-β 1a SC	REL	D, MRI, PROM, UE	[29]
2011	2018	PPMS	Ocrelizumab (Ocrevus®)	120 weeks, N 732	ORATORIO	Placebo	D	O, MRI, PROM, UE	[30]
Unknown	1998	RRMS	Interferon-β1a SC (Rebif®)	48 weeks, N 677	EVIDENCE	Interferon-β-1a IM	REL	MRI, O, UE	[1, 9]
Unknown	2003	SPMS	Mitoxantrone (Ralenova®)	104 weeks, N 194	MIMS	Placebo	REL, D, O	UE, PROM	[1, 12]

*D* disability progression (including EDSS score, EDSS progression), *MRI* MRI outcomes (including T1, T2 lesions, gadolinium-enhancing lesions, brain volume), *PROM* patient-reported outcome measures (including symptoms or quality of life, patient-related outcome such as fatigue), *REL* relapses (including relapse rate, relapse risk, annualized relapse risk), *O* other (including at least one MS-related admission to hospital, at least one MS-related steroid course, time to clinically definite MS, time to McDonald MS), *UE* undesired endpoints

^a^According to Teva product characteristics “Copaxone® 20 mg/ml”, status July 2018, indicated for the treatment of relapsing multiple sclerosis

All drugs licensed to date were tested in 1- to 2-year (rarely longer) pivotal trials, mostly against placebo [20], although more recently, active comparators have also begun to be applied. From the patient’s point of view, some of the primary and secondary endpoints of these studies have limited relevance [27, 65]. Moreover, methodologically sound data on these drugs’ efficacy and safety (or detrimental effects), beyond the duration of these trials, are practically non-existent. The little data covering 3 years or more of application mostly derive from “extension studies” to initial phase III studies or from registers such as “MSBase” [36]. Specialized statistical analyses are applied to compensate for the poor methodological quality of “observational studies” in order to gain insight into the efficacy of immunomodulatory treatments (including compared with each other). However, the “real-world” data gathered in registers are generally not suited for such analyses [31]. Overall, these factors suggest a general approach to designing clinical MS trials that leaves room for improvement and which has hampered our understanding of the long-term benefits and risks of disease-modifying MS treatment. However, these deepened insights are urgently needed to enable neurologists to proceed from a mere “relapse-preventative” strategy when prescribing immunotherapies towards provision of personalized medical services that take the multiple facets of the disease and patient preferences into consideration [22, 45] and also adopts the aim of targeted prevention of adverse events.

## Investigative goal

The goal of this study is, firstly, to set out an overview of the primary and secondary endpoints of pivotal phase III trials in MS. Secondly, based on this summary, as well as our analysis of the shortcomings of clinical trial design to date, we propose a number of suggestions for improvement. Here, we also draw on the latest insights into MS pathophysiology, as well as aspects relevant for patients, particularly the implementation of “patient-reported outcome measures” (PROM). Moreover, we describe the ongoing, significant demand for trials with therapeutic agents that modify disease progression, for which there have been too few controlled studies to date.

## Materials and methods

Our research of the available literature yielded a systematic overview of published pivotal phase III MS trials performed to provide evidence for drug marketing approval (so-called pivotal trials). We took as a starting point an assessment of 21 randomized, controlled phase III trials on relapsing-remitting multiple sclerosis (RRMS) presented in Torkildsen et al. [74]. As all of the latter were completed prior to May 21, 2015, we augmented them with our own research into the literature, focusing on further *completed and published phase III MS trials* (inclusion criteria), as well as extending analysis of all the included trials to the disease courses relapsing multiple sclerosis (RMS), primary progressive MS (PPMS), secondary progressive MS (SPMS), and clinically isolated syndrome (CIS). Drugs not approved for the market despite phase III trial were not included (exclusion criteria). The literature was searched using PubMed, as well as the European public assessment reports (EPAR) of the European Medicines Agency (EMA) and the dossier assessments of early benefit assessments conducted by the German Institute for Quality and Efficiency in Health Care (IQWiG). The PubMed search was conducted using the keywords *Multiple Sclerosis*, *Phase 3*, *trial,* with last access on 2-15-2019.

The following characteristics of the phase III trials were analyzed: trial duration, sample size, comparator drugs (primary, secondary, MRI (magnetic resonance imaging) outcomes, as well as patient-reported outcome measures (PROM)).

## Results

Table 1 of the Appendix summarizes the characteristics of the 29 assessed pivotal phase III trials. Below we describe the key results of our investigation of the trials for the disease courses RRMS, RMS, SPMS, PPMS, and CIS.

### Trial duration

The analysis showed that the phase III RRMS trials conducted since the 1990s had a duration of approximately 2 years, with some few exceptions (e.g., EVIDENCE trial, interferon beta-1a, 1-year duration). For RMS, the trial duration was also generally 2 years. Exceptions here were the 1-year TRANSFORMS trial (fingolimod, approved 2011) and the TOWER trial (teriflunomide, approved 2013), which had a variable duration, but was already completed 48 weeks after inclusion of the last patient. Alone, the PRECISE trial (glatiramer acetate for CIS, approved 2001) had a study duration of 3 years. Only recently have trials longer than 2 years been carried out, including the DECIDE trial (daclizumab for RRMS, 144 weeks, approved 2016, market withdrawal 2018) and the ORATORIO trial in PPMS (ocrelizumab, 120 weeks, approved 2018).

### Number of participants in MS pivotal trials

In recent years, the number of trial participants has increased significantly. While the first pivotal interferon beta 1b trial MSSG only included 372 patients, the DECIDE trial (daclizumab) recruited 1841 patients. One of the reasons for this is that relapse rates have decreased significantly over the last 20 years, for instance because many patients are already being treated with immunomodulatory agents and therefore patients with milder disease course are more likely to be recruited for drug trials. Thus, today significantly higher case numbers are needed to reach statistical significance using annual relapse rate as primary endpoint, with absolute differences between investigational medicinal product (IMP) and comparator drug sometimes averaging just < 0.2 relapses per year. Miniscule effects that only reach statistical significance by inflation of sample size suggests that such trial results are of questionable clinical relevance. However, it should be noted that one advantage of larger sample sizes is a greater chance of detecting rare side effects.

### Comparator drugs

The earliest RRMS trials tested the IMP against placebo as no other immunomodulatory agents had yet been developed. However, more recently, trials are increasingly carried out against active comparators, such as against interferon beta-1a in the RRMS trials CARE MS-1 (alemtuzumab), DECIDE (daclizumab), and EVIDENCE (SC vs. IM interferon beta-1a). Glatiramer acetate was used as comparator drug in the BEYOND trial (interferon beta-1b). In RMS, seven placebo-controlled trials and four trials (TRANSFORMS (fingolimod), CARE MS-2 (alemtuzumab), TENERE (teriflunomide), and OPERA I + II (ocrelizumab)) with interferon beta-1a as active comparator were carried out. In the RMS trial REGARD (interferon beta-1a) glatiramer acetate served as active comparator. The cytotoxic agent mitoxantrone for SPMS was tested against placebo in the MIMS trial, as was the monoclonal antibody ocrelizumab for PPMS in the ORATORIO trial. All four trials in CIS were also placebo-controlled: CHAMPS (interferon-β1a), BENEFIT (interferon-β1b), PRECISE (glatiramer acetate), and REFLEX (interferon-β1a).

### Endpoints

In RRMS trials, relapse rate was most frequently selected as primary endpoint. Disability progression, measured according to the “Expanded Disability Status Scale (EDSS)” for the quantification of neurological disability and confirmed after 12 or 24 weeks, was selected as primary endpoint in two trials (interferon-β1a, MSCRG, and alemtuzumab, CARE MS 1), but served only as secondary endpoint in most. Apart from adverse events, key secondary or explorative endpoints included MRI endpoints, such as number and volume of gadolinium-enhancing lesions and T2-hyperintense or T1-hypointense lesions in cranial MRI and, most recently, also the progression of cerebral atrophy [61]. For the 12 RMS trials, only relapse rate was selected as primary endpoint, albeit in the case of alemtuzumab (CARE MS 1 trial) in combination with disability progression. The pattern was similar for clinically isolated syndrome (CIS): the primary endpoints of the PRECISE trial (glatiramer acetate) were the rate of conversion to clinically definite MS as defined by a second clinical event, while the BENEFIT trial (interferon-β 1b) measured conversion to both clinically definite MS and McDonald MS, as well as the annual relapse rate and the degree of disability. Apart from the primary endpoint (“disability progression confirmed at 12 weeks”), the PPMS trial on ocrelizumab (ORATORIO trial) also investigated secondary endpoints such as “disability progression confirmed at 24 weeks”, MRI endpoints, as well as a patient-reported outcome (quality of life according to the SF-36 (Short Form (36) Health Survey)).

### Patient-reported outcome measures (PROM)

In many cases, patients and physicians differ in the importance ascribed to particular symptoms and consequences of the disease [27, 65]. In general, patients tend to focus far more on disability progression impacting quality of life, rather than disease progression as measured by anatomical, biological, and clinical data. As such, patients generally understand disease progression as the worsening of symptoms, with fatigue, depression, cognitive impairment, pain, spasticity, sleep disturbance, loss of visual functioning, and mobility among those considered most burdensome [16, 17, 24, 27, 57, 60, 63, 65, 77, 78, 80]. Many of these symptoms can be easily quantified using internationally established and validated patient questionnaires.

Virtually, no drug approval trial has systematically investigated PROM. Where investigated, the focus is on fatigue, which is considered by many MS patients to be one of the most troubling symptoms [79]. Here, two examples are the TENERE and TEMSO trials (both teriflunomide, RMS), which investigated fatigue as secondary endpoint using the “Fatigue Impact Scale (FIS)”. In the ocrelizumab trials (OPERA I, OPERA II, ORATORIO), health-related quality of life (HRQoL) was measured using the established, generic survey SF-36, which comprises the section vitality, physical functioning, bodily pain, general health perceptions, physical role functioning, emotional role functioning, social role functioning, and mental health. Nevertheless, measuring HRQoL is far from standard in clinical MS trials, as evidenced by the CLARITY trial for the oral drug cladribine [34], which did not include any PROM parameters [21]. Overall, PROM are still investigated less frequently as primary or secondary endpoints than relapse rate, disease progression, or MRI parameters.

## Discussion

### Deficits in the design of phase III trials to date

Systematic analysis of the phase III trials included in this overview showed that the approval of new substances for the treatment of multiple sclerosis were as a rule randomized, controlled studies of at least 1 year and each included several hundred, sometimes over 1000, patients. This, in principle, suggests that an established approach to designing MS clinical trials exists to a greater extent than in other neurological disorders. However, on closer inspection, it becomes clear that our approach to MS clinical trial design urgently needs to redirect focus towards patient needs, as opposed to biological indicators and surrogate measures of dubious clinical importance.

In the EMA’s “Guideline on clinical investigation of medicinal products for the treatment of Multiple Sclerosis” [30], relapse rate and disability progression are singled out as the most important primary endpoints. The guideline distinguishes between the “accumulation of disability” in terms of relapse rate in RMS and disability progression in SPMS or PPMS in phase III trials, with clinically measured prevention or delay of disability progression recommended as primary endpoint for SPMS and PPMS. For patients with RRMS or SPMS with relapsing MS (RMS), both relapse rate and the time to relapse are accepted as primary outcomes. Relapse rate, or that is, the proportion of relapse-free patients along with the progression of disability should, in addition to MRI outcomes, be investigated as secondary endpoints, insofar as they have not already been examined as primary endpoints. Furthermore, the EMA guideline calls for more emphasis on PROM, as symptoms such as subjective visual function, pain, bladder control, depression, sleep disorder, fatigue, and cognitive dysfunction are enormously important for quality of life and are considered more crucial by some patients than purely somatic outcomes [26, 65].

Analysis of the phase III trials to date highlights significant deficits in pivotal MS trials. These include a treatment duration that is often too short, discrepancies between the hypothesized mechanism of action and inclusion criteria (inclusion of patients with little disease activity or very long disease duration in trials with substances that have strong anti-inflammatory effect), premature confirmation of disease progression (already after 12 weeks), or the lack of relevant PROM. The attempt to obtain statistical significance with high patient numbers despite often only minimal absolute differences in the relapse rate between IMP and comparator drug are both of questionable clinical relevance and of dubious cost-effectiveness (insofar as these resources are then not available for other trials).

Investigating outcomes that are particularly important to patients has been the exception in drug approval trials to date and where the case, they only serve as secondary or explorative endpoints. These approaches are methodologically inadequate to some extent, for example because the PROM explorative endpoints were usually not surveyed using validated measurement instruments (e.g., measurement of fatigue in the TRANSFORMS trial on fingolimod with the unvalidated questionnaire “draft 39-item version of the U-FIS”) [35].

### Suggestions for improving phase III MS trial design

The hypothesized mechanism of action should be clearly described at the beginning of the trial and should be taken into consideration when designing the study. For immunomodulatory drugs intended for treatment in (highly) active disease stages (e.g., natalizumab, ocrelizumab), this would mean that relapse rate could continue to serve as endpoint. However, aspects such as the severity of the relapse or the functional disability and the remission should also be taken into account. To establish the added benefit of a new drug, a clinically significant effect on functionally debilitating relapses (e.g., visual functioning, mobility, physical strength) should be demonstrated. As the timing of relapses is difficult to predict, the duration of observation in trials that take relapse rate as endpoint should be at least 2 years.

For substances with a hypothesized effect on disability progression, the observation period should be of suitable duration (at least 3 years, ideally up to 5 years). Currently, disability progression is commonly established after only 12 or 24 weeks. However, recovery from relapse can take up to 6 or even 12 months, and temporary disability changes stemming from previous relapses can lead to overestimation of long-term disability progression. Consequently, disability progression should only be confirmed after 12 months, which reduces confounding effect of incomplete recovery from recent relapses [42].

The inclusion criteria for the trial subjects should take into account the expected effect of the active substance and be compatible with the primary endpoint. For example, validation of a drug with hypothesized effect on disability progression should include patients whose progression is confirmed prior to inclusion in the trial. Otherwise, the danger exists of including significant numbers of “stable” patients or those with only slow progression. In such cases, the drug being tested might indeed have an effect in the subpopulation, but this might not be detected in the sample due to the mild natural history of the cohort (false negative result for the subpopulation in question). Conversely, a “positive” effect could be the result of the actual subpopulation of interest, but be mistakenly extended to include patients with mild natural history (false positive result for the subpopulation with mild natural history, see ORATORIO trial, ocrelizumab).

For drugs with strong anti-inflammatory effect, focus should be placed on including patients in early disease phases with higher disease activity, instead of—as was most recently the case in the CLARITY trial—a very broad enrolment of the population, including patients with low disease activity and extremely long disease duration. By ensuring the inclusion criteria is compatible with the hypothesized mode of action, it might be possible to achieve clinically relevant results with smaller case numbers, thereby sparing patients the risks involved in testing new immunotherapies. The subjects should also cover a wide age range (up to 60 or 65 years, as opposed to the currently usual age limit of 55) and age effects should be investigated, as a recent meta-analysis demonstrated that age can affect the efficacy of an immunomodulatory therapy [82]. Apart from EDSS, it is imperative that a functional test of low-contrast sensitivity (e.g., low contrast letter acuity using Sloan charts) and mobility (e.g., 6-min walk test) be performed to quantify disability, as vision and mobility are the most important bodily symptoms from a patient point of view [27].

The EMA guideline [30] recommends evaluating health-related quality of life, although lack of data precludes recommending any specific instruments. Indeed, using established assessment instruments to quantify health-related quality of life, as well as fatigue and cognition, should become standard practice. Tools for measuring quality of life include MSQOL 54 (Multiple Sclerosis Quality of Life-54) [38], HAQUAMS (Hamburg Quality of Life Questionnaire in Multiple Sclerosis) [66], MSQLI (Multiple Sclerosis Quality of Life Inventory) [39], or the recently developed Neuro-QoL [51]. Fatigue and cognition can be measured using the Fatigue Severity Scale (FSS) [37, 46] and BICAMS (Brief International Cognitive Assessment for Multiple Sclerosis) [32, 48], respectively. A simple further screening instrument for cognition is the SDMT (Symbol Digit Modalities Test) [75]. The effect of limited vision and mobility on quality of life should be quantified using the established measurement instrument NEI-VFQ25 (National Eye Institute Visual Functioning Questionnaire 25) [40] or the MSWS-12 [23], as applies.

Cerebral or spinal MRI parameters can serve as secondary or explorative outcomes (e.g., T2 lesions, spinal cord atrophy [2, 83]); however, the repeated application of gadolinium-based MRI contrast agents should be avoided due to safety concerns [14, 67]. Moreover, brain atrophy measurements, although technically feasible in a clinical study with rigorous standardization of assessments, are not recommended as they are not yet transferable to gauge prediction and monitoring of disease course in individual patients, thus currently not supporting personalization of medical services [3, 28, 61, 62, 81]. The same applies to other advanced imaging modalities such as diffusion tensor imaging, ultrahigh field MRI and others [47, 56, 59, 64, 69, 70, 72]. Most recently, the use of retinal optical coherence tomography (OCT) for the quantification of axonal and neuronal damage caused by MS is increasing [4, 10, 50, 52–54, 84]. This technique has been occasionally used as outcome measure in clinical trials and might serve as predictive diagnostics for disease course and response to immunotherapy both in trial cohorts and individual subjects in the future. A further suitable secondary or explorative outcome that might be established in the near future both for clinical studies and individualized prediction is the identification of neurofilaments in serum as surrogate marker of axonal damage in the CNS [1, 6, 7, 12, 43, 49, 73, 76].

Exclusively placebo-controlled trials with a duration of more than 6 months that test disease-modifying drugs in RRMS are ethically problematic. Moreover and importantly, the advantages and disadvantages of individual treatment options cannot be identified by means of placebo-controlled studies. When selecting appropriate active comparators, care should be taken that the trial’s inclusion criteria reflect both the study population and the active comparator’s approval status. In trials with a PPMS population, the lack of approved drugs (with the exception of ocrelizumab) justifies the use placebo-controlled trials. Ocrelizumab, which was recently approved, likely only benefits a small subgroup of younger PPMS patients (up to 45 years) with short disease duration (up to 15 years) and disease activity in MRI (new T2 lesions, gadolinium-enhancing lesions), and trialing against this substance in other PPMS populations (older patients, longer disease duration, no MRI activity) makes little sense and is moreover not ethically acceptable.

Overall, the high demand of patients for studies in progressive MS (SPMS, PPMS) continues. A significant number of drugs, including some approved for treatment of RRMS, were unsuccessfully tested in patients with progressive MS. The reasons for this are manifold and include an incomplete understanding of the pathophysiology of progression, an insufficiently detailed grasp of the mechanism of action of the drug, and the shortcomings of the applied outcomes (such as the EDSS with high inter-rater variability and disproportionate weighting allocated to lower extremity functioning, or more generally, the ability to walk).

The suggestions for improvement presented here build to some extent on those recently published by Ontaneda et al. ([55], see Text Box 5b). Importantly, clinical researchers planning a trial should make use of the EMA’s advice service, and ideally also involve the European HTA (Health Technology Assessment) institutions [29], of which the Federal Joint Committee (G-BA) is the primary body in Germany [33]. The aim of the consultation process should be ensuring that the trial is designed to not only meet the requirements necessary for market approval, but also to inform treatment decisions and to perform an assessment of the added benefit of the new drug compared to standard treatment. The results of the consultation should be published to ensure transparency.

Pharmaceutical companies in Germany sometimes complain that the recommendations of drug approval agencies and HTA institutions (IQWiG and G-BA) diverge to some extent and that data from a single phase III trial is interpreted differently by the EMA, on the one hand, and the committees participating in the early benefit assessment (IQWiG and G-BA), on the other. However, this perspective does not take into account the fact that the questions posed by the approval agencies (“Does the new substance have a positive benefit-risk balance?”) and the benefit assessment (“Does the new substance have an added benefit compared to a ‘standard therapy’?”) are very different. By necessity, this leads to diverging demands on the trial design in the first instance, and ultimately leads to different interpretation of the obtained results in the final instance.

Despite our criticism of MS studies to date, we are aware that planning a clinical trial and improving quality and patient focus as discussed above also (have to) take into account a wider health policy and regulatory framework, as well as financial considerations. This complicates and could even hobble design of a study that focuses exclusively on achieving scientific insights into MS.

### Expert recommendations

Fortunately, the therapeutic landscape for people with MS has significantly broadened over the past 15 years. In parallel to the increasing number of available immunotherapies, treatment strategies in MS have shifted from a mere “relapse-prevention” approach to a personalized provision of medical care as to the choice of the appropriate drugs and their sequential application over the course of the disease. This personalized provision should take patient preferences as well as disease-related factors into consideration such as objective clinical and radiographic findings but also very burdensome symptoms such as fatigue, depression, and cognitive impairment. This change in perspective on what physicians want to accomplish for their patients has not only been endorsed by clinicians and researchers but has also been adopted by regulatory bodies such as EMA. Therefore, future trial designs in MS should assign higher relevance to these patient-reported outcomes and should aim at implementing measures that can serve as predictive markers for individual treatment response to new and investigational immunotherapies. This is an indispensable prerequisite to maximize the benefit of individual patients when participating in clinical trials and when starting on an immunotherapy post approval. Moreover, such appropriate trial designs and suitable enrolment criteria that correspond to the mode of action of the study drug will facilitate targeted prevention of adverse events, thus mitigating risks for individual study participants. Finally, personalized provision of medical services prior to enrolment into clinical trials must encompass utmost accuracy when diagnosing MS and ruling out relevant differential diagnoses given that newer and highly efficacious immunotherapies for MS might cause harm in other MS mimics such as neuromyelitis optica spectrum disorders and many others [5, 8, 9, 11, 15, 18, 19, 41, 44, 53, 68, 71].

5a Text box “Suggestions for improving the design of (R)RMS^1^/CIS trials”

**Table:** 

Duration: Not less than 2 years, active comparator Outcomes: Relapse rate, only functionally debilitating (relapse involving EDSS worsening of at least 1 point) relapses with full or partial recovery should be included Disability progression: in RRMS trials involving patients with short disease duration, it makes little sense to investigate after 12 or 24 weeks; instead the number of functionally debilitating relapses and their remission should be examined; comparing disability at the end of a 2-year trial compared to baseline recommended Additional for CIS: Time to conversion to CDMS (Clinically Definite Multiple Sclerosis) Further outcomes/measurement instruments: - General: EDSS, MSFC (Multiple Sclerosis Functional Composite) - Vision: Snellen Visual Acuity Test and LCLA (Low Contrast Letter Acuity), e.g., using Sloan charts) - Mobility: 6-min walk test - Cognition: BICAMS or at a minimum SDMT - PROM: Fatigue (FSMC (Fatigue Scale for Motor and Cognitive Functions)) or FSS), depression BDI-II (Beck Depression Inventory II); general quality of life: HAQUAMS (Hamburg Quality of Life Questionnaire in Multiple Sclerosis), SF36 or MSQoL; vision-related quality of life: NEI-VFQ25; mobility-related quality of life: MSWS-12 (Multiple Sclerosis Walking Scale); sleep-related quality of life: PSQI (Pittsburgh Sleep Quality Inventory); pain-related quality of life: Brief Pain Inventory (BPI) - MRI: Repeated administration of gadolinium-based contrast agent to be avoided, T2 lesions recommended, brain atrophy not recommended as clinical value unclear, spinal cord atrophy as potentially promising imaging marker - OCT (Optical Coherence Tomography) with GCIPL (Ganglion Cell-inner Plexiform Layer) and RNFL (Retinal Nerve Fiber Layer)	

5b Text box “Suggestions for improving the design of PPMS/SPMS^2^ trials”

**Table:** 

Prerequisite: Inclusion only of patients with proven clinical progression PRIOR to inclusion (e.g., at least 1 EDSS point in prior 1–2 years) Duration not less than 3 years, preferably up to 5 years, placebo may need to be justified Endpoints: Confirmed disability progression after 12 months Further endpoints/measurement instruments: - General: EDSS, MSFC (Multiple Sclerosis Functional Composite) - Vision: Visual Acuity Test and LCLA (Low Contrast Letter Acuity), e.g., using Sloan charts) - Mobility: 6-min walk test - Cognition: BICAMS or at a minimum SDMT - PROM: Fatigue (FSMC (Fatigue Scale for Motor and Cognitive Functions)) or FSS), depression BDI-II (Beck Depression Inventory II); general quality of life: HAQUAMS (Hamburg Quality of Life Questionnaire in Multiple Sclerosis), SF36, MSQoL or Neuro-QoL; vision-related quality of life: NEI-VFQ25; mobility-related quality of life: MSWS-12 (Multiple Sclerosis Walking Scale); sleep-related quality of life: PSQI (Pittsburgh Sleep Quality Inventory); pain-related quality of life: Brief Pain Inventory (BPI) - MRI: Repeated administration of gadolinium-based contrast agent to be avoided, T2 lesions recommended, brain atrophy not recommended as clinical value unclear, spinal cord atrophy as potentially promising imaging marker - OCT (optical coherence tomography) with GCIPL (ganglion cell-inner plexiform layer), and RNFL (retinal nerve fiber layer)	

## Footnotes

1 RMS also includes patients with SPMS and relapse activity

2 Particularly patients without relapse activity

## Abbreviations

AMNOG: The Act on the Reform of the Market for Medical Products
BDI-II: Beck Depression Inventory II
BICAMS: Brief International Cognitive Assessment for Multiple Sclerosis
BPI: Brief Pain Inventory
CDMS: Clinically definite multiple sclerosis
CIS: Clinically isolated syndrome
EDSS: Expanded Disability Status Scale
EMA: European Medicines Agency
EPAR: European Public Assessment Reports
FIS: Fatigue Impact Scale
FSMC: Fatigue Scale for Motor and Cognitive Functions
FSS: Fatigue Severity Scale
G-BA: Federal Joint Committee
GCIPL: Ganglion cell-inner plexiform layer
HAQUAMS: Hamburg Quality of Life Questionnaire in Multiple Sclerosis
HTA: Health technology assessment
IQWiG: German Institute for Quality and Efficiency in Health Care
LCLA: Low Contrast Letter Acuity
MRI: Magnetic resonance imaging
MS: Multiple sclerosis
MSFC: Multiple Sclerosis Functional Composite
MSWS-12: Multiple Sclerosis Walking Scale
MSQLI: Multiple Sclerosis Quality of Life Inventory
MSQOL-54: Multiple Sclerosis Quality of Life-54
NEI-VFQ25: National Eye Institute Visual Functioning Questionnaire 25
Neuro-QoL: Quality of Life in Neurological Disorders
OCT: Optical coherence tomography
PPMS: Primary progressive multiple sclerosis
PROM: Patient-reported outcome measure
PSQI: Pittsburgh Sleep Quality Inventory
RMS: Relapsing multiple sclerosis
RNFL: Retinal nerve fiber layer
RRMS: Relapsing-remitting multiple sclerosis
SDMT: Symbol Digit Modalities Test
SF36: 36-Item Short Form Survey
SPMS: Secondary progressive multiple sclerosis
